# Transmission of Predictable Sensory Signals to the Cerebellum via Climbing Fiber Pathways Is Gated during Exploratory Behavior

**DOI:** 10.1523/JNEUROSCI.0439-16.2016

**Published:** 2016-07-27

**Authors:** Charlotte L. Lawrenson, Thomas C. Watson, Richard Apps

**Affiliations:** ^1^School of Physiology, Pharmacology, and Neuroscience, University of Bristol, Bristol BS8 1TD, United Kingdom,; ^2^Neuroscience Paris Seine, Cerebellum, Navigation, and Memory Team, Sorbonne Universities, Université Pierre et Marie Curie, University of Paris 06 Unité Mixte de Recherche Scientifique 8246, INSERM Unité Mixte de Recherche Scientifique 1130, and Centre National de la Recherche Scientifique Unité Mixte de Recherche 8246, F-75005 Paris, France

**Keywords:** cerebellum, exploratory, inferior olive, learning, motor behaviour, sensory

## Abstract

Pathways arising from the periphery that target the inferior olive [spino-olivocerebellar pathways (SOCPs)] are a vital source of information to the cerebellum and are modulated (gated) during active movements. This limits their ability to forward signals to climbing fibers in the cerebellar cortex. We tested the hypothesis that the temporal pattern of gating is related to the predictability of a sensory signal. Low-intensity electrical stimulation of the ipsilateral hindlimb in awake rats evoked field potentials in the C1 zone in the copula pyramidis of the cerebellar cortex. Responses had an onset latency of 12.5 ± 0.3 ms and were either short or long duration (8.7 ± 0.1 vs 31.2 ± 0.3 ms, respectively). Both types of response were shown to be mainly climbing fiber in origin and therefore evoked by transmission in hindlimb SOCPs. Changes in response size (area of field, millivolts per millisecond) were used to monitor differences in transmission during rest and three phases of rearing: phase 1, rearing up; phase 2, upright; and phase 3, rearing down. Responses evoked during phase 2 were similar in size to rest but were smaller during phases 1 and 3, i.e., transmission was reduced during active movement when self-generated (predictable) sensory signals from the hindlimbs are likely to occur. To test whether the pattern of gating was related to the predictability of the sensory signal, some animals received the hindlimb stimulation only during phase 2. Over ∼10 d, the responses became progressively smaller in size, consistent with gating-out transmission of predictable sensory signals relayed via SOCPs.

**SIGNIFICANCE STATEMENT** A major route for peripheral information to gain access to the cerebellum is via ascending climbing fiber pathways. During active movements, gating of transmission in these pathways controls when climbing fiber signals can modify cerebellar activity. We investigated this phenomenon in rats during their exploratory behavior of rearing. During rearing up and down, transmission was reduced at a time when self-generated, behaviorally irrelevant (predictable) signals occur. However, during the upright phase of rearing, transmission was increased when behaviorally relevant (unpredictable) signals may occur. When the peripheral stimulation was delivered only during the upright phase, so its occurrence became predictable over time, transmission was reduced. Therefore, the results indicate that the gating is related to the level of predictability of a sensory signal.

## Introduction

The inferior olive climbing fiber (olivocerebellar) projection is essential for normal cerebellar operation, but the information conveyed by these afferents remains a subject of intense debate. Olive cells and the complex spikes they generate in Purkinje cells are highly sensitive to stimulation of their peripheral receptive field when an animal is at rest but do not respond reliably during active movement (Gellman et al., 1985; Andersson and Armstrong, 1987). Such findings have led to the notion that transmission in spino-olivocerebellar pathways (SOCPs) is “gated,” thereby limiting the times when sensory signals from the periphery can be forwarded to the cerebellum by this route (Gellman et al., 1985; Kim et al., 1987; Wang et al., 1987; Horn et al., 1996; Apps, 1999). This in turn imposes constraints on the signaling capabilities of climbing fibers and therefore provides important clues to their function.

For example, transmission in SOCPs relaying signals to the “forelimb-receiving” parts of the cerebellar C1 and C3 zones in lobule V of the cat cerebellum is increased during the swing phase of the ipsilateral forelimb step cycle and reduced during stance (Lidierth and Apps, 1990; Apps et al., 1995; Apps and Lee, 1999; Pardoe et al., 2004). The swing phase is the time during locomotion when the limbs are most likely to encounter obstacles to progression, whereas the stance phase is when self-generated (reafferent) sensory signals are likely to occur as a result of load bearing. Therefore, the gating in SOCPs that target the C1 and C3 cerebellar cortical zones may serve to gate out the predictable (internally generated) sensory consequences of a movement, while permitting transmission of unpredictable (externally generated) sensory signals that can be used to update an internal model (Wolpert and Miall, 1996).

If this is the case, then the pattern of gating in SOCPs should be modifiable if a sensory stimulus becomes predictable. Some evidence to support this possibility has been obtained during motor learning (Sears and Steinmetz, 1991; Hesslow and Ivarsson, 1996; Apps and Lee, 2002). However, these previous studies involved classical conditioning of an eyeblink or forelimb-withdrawal reflex (associative learning of a simple motor response), and it remains unknown whether the gating is modifiable during voluntary movement. Hence, the current experiments developed a behavioral model in rats that takes advantage of their exploratory activity of rearing. Rearing involves a rat using its hindlimbs to stand upright to scan the environment. During rearing, a rat is vulnerable to predator attack, so it is important that the animal is able to respond at this time to any unexpected peripheral sensory events, including external stimuli to the hindlimbs.

Previous studies of gating of transmission in SOCPs during movement have focused on forelimb pathways (Apps, 1999). Given that hindlimb paths have a different pattern of organization in terms of number and location of central sites of synaptic relay and differ in their functional responsibilities, it is an open question whether they are also subject to modulation during movement and, if so, what the pattern of gating might be. We first investigated the pattern of gating of transmission in hindlimb SOCPs in relation to rearing behavior and then determined whether a peripheral stimulus, when delivered repeatedly at the same time during rearing, can modify the pattern. When the peripheral stimulus was delivered unpredictably to the hindlimb, the largest reductions in transmission relative to rest occurred during rearing up and rearing down, which coincide with active movement (when self-generated afference is likely to occur), whereas transmission was similar to rest during the upright phase of rearing. When the hindlimb stimulation was delivered only during the upright phase of rearing, so its occurrence became predictable over time, there was a progressive reduction in pathway transmission. Therefore, the findings demonstrate that gating of hindlimb SOCPs occurs during exploratory behavior in rats, can be modified by experience, and is related to the level of predictability of the sensory signal.

## Materials and Methods

### 

#### Animals

All animal procedures were performed in accordance with the United Kingdom Animals (Scientific Procedures) Act 1986 and local ethical guidelines. Experiments were performed on 10 adult male Wistar rats (300–450 g; Charles River). Animals A–J are identified as GR(A–J) throughout. All animals were housed under normal environmental conditions (∼20°C and 45–65% humidity) on a reverse 12 h dark/light cycle and provided with food and water *ad libitum*.

#### Implants

For chronic and subsequent acute experiments (see below), miniature microdrives (weight of ∼2.5 g) were built in house, each incorporating up to four independently movable electrodes (two to four bundles of 12.5 μm tungsten wire for multitrodes or 50.8 μm insulated stainless steel wire for single microwires; impedance of 80–400 kΩ at 1 kHz, all from California Fine Wire). Aseptic surgery was performed under sodium pentobarbital anesthesia (60 mg/kg, i.p.) to expose the paravermal part of lobule V/VI. The microdrive assembly was positioned over the exposure and fixed to the skull with bone screws and dental acrylic (for additional details, see Koutsikou et al., 2015).

During the operation, the recording electrodes were advanced into the cerebellar cortex guided by physiological recording. Percutaneous electrical stimulation (single pulse, 0.2 ms, at a rate of 0.5 Hz) of the ipsilateral hindlimb was used to evoke extracellular field potentials. The recording electrodes were placed at a depth within the cerebellum in which the largest field potentials were evoked. Consistent with previous studies (Atkins and Apps, 1997), these responses had a latency to peak of ∼17 ms (see Results) and were found ∼4 mm from the brain surface.

In six animals, pairs of flexible stainless steel wires (Cooner Wire) were also sutured subcutaneously bilaterally into hindlimb muscles (relating approximately to the biceps femoris and gracilis muscle on opposite sides of the hindlimb) to record electromyography (EMG) activity. In addition, bipolar stimulating wires (Cooner Wire) were sutured subcutaneously within the hindlimb (superficially and in close proximity to the ankle joint) ipsilateral to the cerebellar recording electrodes, and all peripheral leads were fed subcutaneously to connectors within the microdrive headpiece (Pardoe et al., 2004). After recovery, buprenorphine (0.1–0.25 mg/kg Vetergesic multidose) or carprofen (2–5 mg/kg Rimadyl) was given subcutaneously as an analgesic. After surgery, animals were individually housed under normal environmental conditions as above.

#### Chronic recording

After recovery from surgery, differential recordings were made using a Lynx 8 system (Neuralynx), CED 1401 A/D device, and Spike 2 acquisition software (Cambridge Electronic Design). A contralateral skull screw above the cerebellum served as the reference for cerebellar field potential (CFP) signals. EMG recordings from either side of the neck or hindlimb were referenced against each other. Both EMG and CFP signals were sampled at 5 kHz and bandpass filtered (0.1 Hz to 1 kHz). Single-unit activity within the cerebellar cortex was sampled at 25 kHz and bandpass filtered (300 Hz to 6 kHz). Video recordings were made throughout the recording sessions using two USB web cameras (30 frames/s capture rate). The cameras were positioned to the side and angled above the home cage and rearing box. Video images were synchronized with electrophysiological data in Spike 2 software. All data analysis was performed in Spike 2 version 7 (Cambridge Electronic Design) and all statistical tests within Prism version 5 (GraphPad Software). All data were tested for a Gaussian distribution. For parameters in which this was not satisfied, the data were transformed logarithmically before parametric statistical analysis was performed or when appropriate nonparametric analysis.

#### Stimulus parameters

During behavior, low-intensity electrical stimuli were delivered via the peripherally implanted stimulating leads (square pulses of 0.2 ms duration; constant-current stimulator DS3; Digitimer) at 1.5 s intervals or triggered in relation to rearing movement (see below). For each animal, the initial recording sessions were used to construct a stimulus response curve to identify the stimulus intensity threshold (T) at which CFPs (evoked responses) could be detected. Incremental increases in stimulus current were delivered, ranging in different experiments from 0.2 to 10 mA. Maximum current intensity was reached when the stimulus evoked a visible twitch from the ipsilateral hindlimb or body. The threshold value was determined when CFP peak-to-peak amplitude was statistically significantly larger than baseline noise levels (calculated using one-way ANOVA with Dunn's multiple comparisons test, *p* < 0.05 was considered to be statistically significant). To construct the average stimulus response curve of all animals combined, the stimulus intensities were expressed as multiples of T. For the behavioral experiments, the stimulus intensity used in different animals varied from 1.1 to 3.1 × T (Table 1).

**Table 1.** Stimulus intensities used in different animals to evoke CFPs during behavior, the duration of the response evoked, and the approximate zebrin band location of individual recording sites in the copula pyramidis

Animal	Current (mA)	Stimulus intensity (T)	Response duration	Approximate recording site location
GRA	3.2	1.5	L	f−/e1+
GRB	6	1.7	S	f−
GRC	4.7	3.1	S	e2−/5+
GRD	2.3	1.9	S	f−/e1+
GRE	1.4	1.8	S	?
GRF	4.4	1.1	S	f−
GRG	2.5	3.1	L	e1+
GRH	3.1	1.7	L	e1−/e2+
GRI	2.5	2.5	S	5+
GRJ	3	2.3	L	e1−/e2+

L, Long duration; S, short duration; ?, histology unavailable.

#### Classifying climbing fiber-evoked fields in the awake rat

##### Paired pulse test.

Paired pulse experiments were performed when rats were sitting quietly at rest in their home cage. Stimuli were applied at ∼2 × T to evoke a CFP with increasing interstimulus time intervals of 30, 60, 90, and 120 ms. The time period between paired stimuli was usually 1.5 s (Pardoe et al., 2004). The intensity of the stimulus typically evoked a mild twitch of the stimulated hindlimb but otherwise did not appear to disturb the animal. The peak-to-peak amplitude of responses was measured, and the mean of the second response was expressed as a percentage of the mean amplitude of the initial response. For group analysis, data were normalized to the maximum response size within each animal.

##### Evoked complex spikes.

To record individual Purkinje cell responses, multitrodes were advanced ventrally into the cerebellar cortex over a number of consecutive days until a single unit was found. To evoke complex spikes, ipsilateral hindlimb electrical stimuli were delivered with the same parameters as detailed above. For data analysis, single units were isolated using principal component analysis in Spike 2 sorting software. Complex spikes were identified and separated from simple spikes by visual identification of their characteristic waveform.

##### Inferior olive lesions.

At the end of the recording period (typically 4–6 weeks after the implant surgery), a terminal experiment was performed in five of the animals under pentobarbitone anesthesia (60 mg/kg, i.p.). The olivary neurotoxin 3-acetylpyridine (3-AP; 75 mg/kg in 1 ml/kg saline; Aldrich) or saline was injected intraperitoneally. CFPs were evoked at ∼1.5 × T once every 3 s, and responses were recorded for up to 4 h. A single-channel data acquisition system (Neurolog) was used in conjunction with a Micro 1401 interface (Cambridge Electronic Design) and Spike 2 version 7 (Cambridge Electronic Design) software to capture evoked and spontaneous local field potential (LFP) data (30 Hz to 5 kHz, gain of 1 k Hz, sample rate of 5 kHz). The peak-to-peak amplitude of responses evoked by 20 stimulus trials were averaged every 30 min to study changes in the size of the evoked CFP over time. The same data were also sampled for 30 s at 30 min intervals to investigate changes in the power spectral density (square millivolts per Hertz) in the 200–300 Hz frequency band of the LFP signal (sampled at 0.5 Hz, frequency resolution of ∼1Hz). Spike 2 software was used to analyze both CFP and LFP data, and the results were normalized to baseline at *t* = −30 min. For LFP data, spectrograms were created using the mtspecgramc.m script from the chronux toolbox in MATLAB (MathWorks; Bokil et al., 2010).

#### Experimental design and analysis during rearing behavior

##### Experiment 1: pseudorandom stimuli.

In all 10 animals, the novel environment of the recording room and removal of the home-cage lid was sufficient to elicit spontaneous rearing activity and bouts of locomotion and rest. Recordings were made for as long as the animal remained active with intermittent bouts of rest (individual recording sessions ranged from 7.6 to 23.5 min). The intensity of the peripheral stimulus was kept constant throughout each recording session (Table 1) and was delivered at a rate of once every 1.5 s. Periods of quiet rest were defined as phase 0, and individual rears were divided into three additional phases: phase 1, rearing up; phase 2, upright; and phase 3, rearing down. Because the peripheral stimulus was delivered independently of the animal's behavior, this meant that the timing of stimuli during each rear occurred pseudorandomly. Typically, only one stimulus was delivered during one phase of each rearing movement but occasionally in two phases. The total number of stimuli delivered throughout each recording session ranged from 253 to 582.

##### Analysis of Experiment 1.

For each rat, the recording session with the largest number of stimulus trials was selected for analysis. The time stamp for each hindlimb stimulation was assigned to one of the following categories of behavior: (1) rest; (2) one of three different phases of rearing (see above); or (3) unclassified movements. For each stimulus trial, various parameters of the evoked CFPs were analyzed. These measurements included onset latency, latency to peak, peak-to-peak amplitude, and area and width of the response. Consistent with previous studies (Apps et al., 1990; Lidierth and Apps, 1990) amplitude and area measurements yielded similar results. Therefore, presentation of results is confined mainly to consideration of area of the evoked CFPs. To aid comparison between animals, the data during rearing were normalized relative to the mean area of the response at rest obtained in the same recording session. Pie charts were also constructed (see Fig. 3) showing for each animal response size evoked during the three different phases of rearing expressed as a proportion of the total percentage change in response size relative to rest. For example, if the responses in phases 1 and 3 were both 50% of mean response size at rest and responses in phase 2 were 100% (giving a total sum change relative to rest of 200%), then the proportions of the pie chart would be 25, 25, and 50%, respectively. For evoked EMG responses recorded in six animals, the onset latency and area of the responses were measured and separated into different phases of rearing as described above. These data were then compared with the CFP responses evoked by the same stimulus.

##### Experiment 2: movement-triggered stimuli.

After obtaining data when the peripheral stimulus was presented pseudorandomly relative to the four different phases of behavior, in three of the animals (GRH, GRI, GRJ), the timing of the stimulus was altered so that it occurred repeatedly during phase 2 of each rear. This was achieved using a “rearing box” built in house (30 × 15 × 30 cm box with clear Perspex walls). An array of infrared emitters and detectors were installed around the top edge of the box to detect when an animal was standing in an upright position (phase 2 of rearing). Disruption of the infrared signal by the animal's upper body during rearing provided a signal (with a delay of ∼4 μs) to trigger the ipsilateral hindlimb stimulation (same parameters as above). The vertical height for both the emitters and detectors was adjusted for individual animals to optimize detection of rearing phase 2. The position of the emitters and detectors was then kept constant over all subsequent recording sessions. Each rat completed two recording sessions per day for 1–2 weeks. The peripheral stimulus was delivered during rearing phase 2 in each recording session. Because natural behavior was being studied, the number of rears varied from day to day, but typically 20 presentations of the stimulus were obtained in each session. During each recording session, responses were also evoked when the animal was sitting quietly at rest (phase 0).

##### Analysis of Experiment 2.

The analysis of the evoked CFPs was the same as for Experiment 1. For every recording session, the mean area of response during phase 2 was expressed as a percentage of the mean area of response evoked at rest during the same recording session and plotted as a function of time.

#### Histology

At the end of every experiment, animals were deeply anesthetized (60 mg/kg, i.p.), and an electrolytic lesion (50–100 μA for ∼5 s) made at cerebellar recording sites. Each animal was perfused transcardially with 4% paraformaldehyde (PFA), and the cerebellum was removed, postfixed in 4% PFA overnight, and histologically processed. Frozen sections were cut at 50–60 μm in the sagittal plane using a microtome (SM 2000R; Leica). Sections were mounted onto glass slides using a 1% gelatin and 0.1% chromium potassium sulfate solution and allowed to air dry overnight. Sections were counterstained with cresyl violet using standard procedures and coverslipped using DPX (Fluka Biochemika). A light microscope (Axioskop 2 plus; Zeiss) was used to identify the location of electrolytic lesions and electrode tracks within the cerebellum. The location of recording sites was matched to sagittal sections of a stereotaxic atlas and then translated to the coronal plane (Paxinos and Watson, 2007). Coronal locations were then compared, using measurements relative to the midline, with zebrin banding patterns described previously (Cerminara et al., 2013).

## Results

### Characterization of hindlimb-evoked climbing fiber field potentials in the awake rat

**Figure 1.**
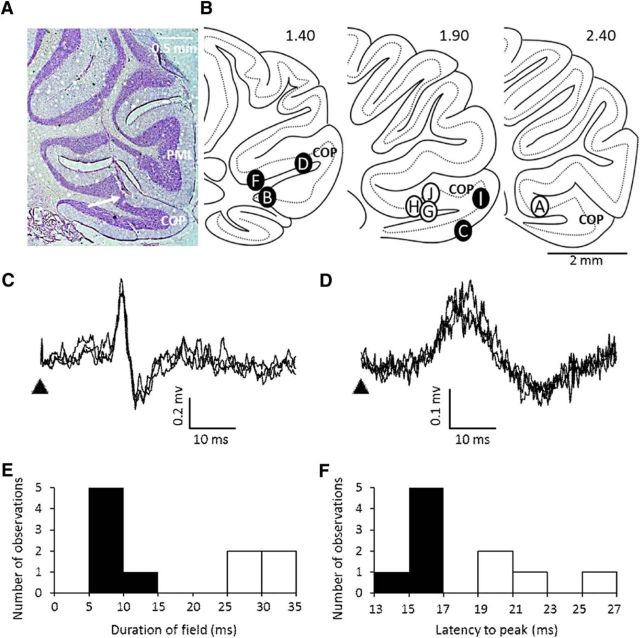
Cerebellar localization and nature of hindlimb evoked fields. ***A***, Example case showing end of electrode track (arrows) within the copula pyramidis (COP). PML, Paramedian lobule. ***B***, Standard sagittal sections of COP (lateral position from midline shown in millimeters) displaying the approximate location of electrode recording sites. Filled circles represent sites in which short-duration responses were obtained, and open circles show site of recording of long-duration responses. All sites are identified with the exception of animal GRE, in which the recording site could not be recovered histologically. ***C***, Example extracellular recordings (3 sweeps superimposed) showing short-duration cerebellar field potential (CFP) evoked by ipsilateral hindlimb stimulation in the awake rat. Arrowhead, Time of stimulus. ***D***, Same as ***C***, but example of a long-duration CFP. Histogram showing the distribution of mean duration of evoked fields (***E***) and the mean latency to peak during rest (***F***) (*n* = 10 rats).

Field potentials evoked by low-intensity ipsilateral hindlimb stimulation were recorded during behavior from the posterior lobe of the cerebellar cortex in a total of 10 adult rats. Postmortem histology in nine cases confirmed that the recording sites were all located within the copula pyramidis (Atkins and Apps, 1997; Fig. 1*A*,*B*). The exception was one animal (rat GRE) in which histological verification of the recording site location could not be obtained because of poor tissue preservation. However, because the responses in this case were indistinguishable from those obtained in the other animals, these data are also included in the analysis.

In six cases, the responses had a short duration of 7.9 ± 2.2 ms (Fig. 1*B*,*C*, filled circles, no histology for one case), whereas in four cases the responses were significantly broader with a duration of 30.0 ± 4.9 ms (Fig. 1*B*,*D*, open circles; unpaired *t* test, *p* = 0.0001; Fig. 1*E*). This difference was also reflected in the latencies to peak of the responses, which were 15.6 ± 0.5 and 21.8 ± 0.3 ms, respectively (unpaired *t* test, *p* = 0.009; Fig. 1*F*). Given the microcircuit organization of the C1 zone in the copula pyramidis and, in particular, the close relationship between cerebellar cortical inputs and outputs and zebrin II bands in this zone (Cerminara et al., 2013), it is possible that the differences in response duration are related to recording site location within different cortical microzonal territories as can be revealed by zebrin bands. For example, recording sites located more medially within the C1 zone may be located in the f− or e1+ zebrin bands (associated with the lateral paw), whereas more lateral recording sites may be situated in the e1− or e2+ bands (associated with the heel area; Cerminara et al., 2013). However, no systematic differences could be found between the two types of response in terms of recording site location and zebrin identity (Table 1), nor any of the other characteristics studied in the present experiments. Therefore, the results for short- and long-duration responses are considered together.

**Figure 2.**
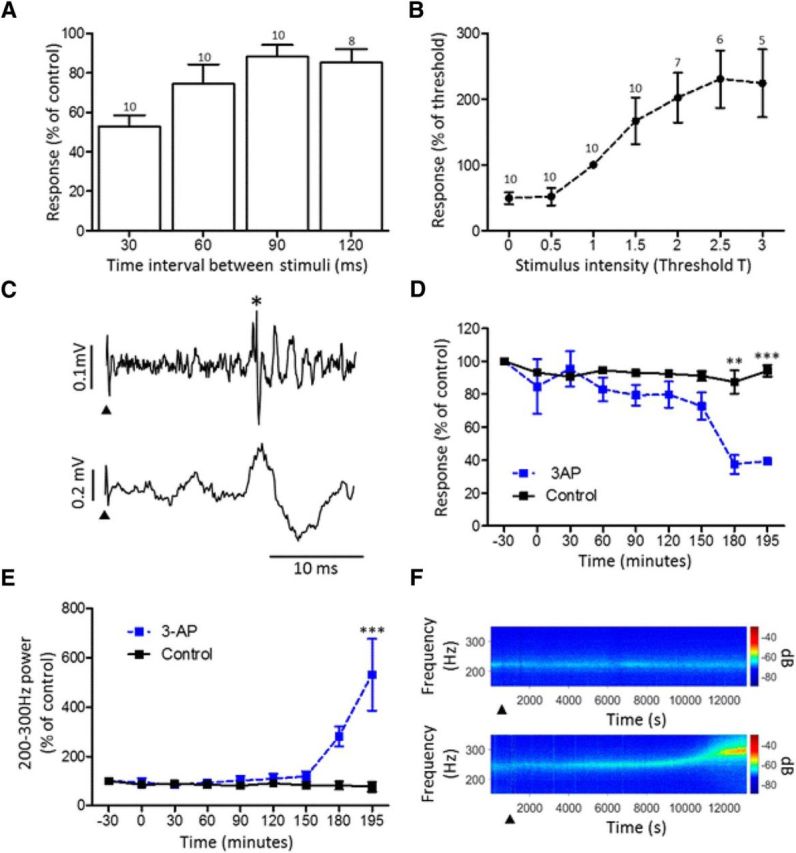
Characterization of evoked CFPs. ***A***, Effect of paired pulse stimulation on the peak-to-peak amplitude of the second response expressed as a percentage of the first control response (mean ± SEM). Number of animals included in each mean shown above each bar. ***B***, Stimulus–response curve for CFPs evoked by ipsilateral hindlimb stimulation at multiples of threshold for a detectable response (mean ± SD). Number of animals included in each mean shown above each data point. ***C***, Example of a complex spike (top trace, asterisk) and CFP (bottom trace) evoked by the same ipsilateral hindlimb stimulation (arrowhead). ***D***, Effect of 3-AP treatment on the amplitude of evoked fields (blue, *n* = 3) compared with saline controls (black, *n* = 2). Two-way ANOVA: interaction, *F*_(8,27)_ = 3.36, *p* = 0.0084; drug, *F*_(1,27)_ = 23.97, *p* < 0.0001; time, *F*_(8,27)_ = 4.59, *p* = 0.0013. Bonferroni's *post hoc* tests comparing 3-AP with control at each time point, **p < 0.01, ***p < 0.001. Drug or saline administered at time 0. ***E***, Effect of 3-AP treatment on 200–300 Hz power of LFP (blue, *n* = 3) compared with saline controls (black, *n* = 2). Two-way ANOVA: interaction, *F*_(8,27)_ = 5.08, *p* = 0.0006; drug, *F*_(1,27)_ = 13.30, *p* = 0.0011; time, *F*_(8,27)_ = 4.55, *p* = 0.0013. Bonferroni's *post hoc* tests comparing 3-AP with control at each time point, ***p < 0.001. Drug or saline administered at time 0. ***F***, Spectrograms derived from an example case (animal GRF) showing change in power in ∼200–300 Hz frequency bandwidth after application of 3-AP (bottom) in contrast to saline (top). Arrowhead, Time of delivery of saline or drug.

On average, the evoked responses in the awake animal had an onset latency of 12.5 ± 0.3 ms (range of 10.9–13.4 ms, *n* = 10). In contrast, under barbiturate anesthesia, responses evoked at the same recording sites had a significantly longer onset latency of 18.75 ± 2.4 ms (Wilcoxon's matched-pairs signed-rank test, *p* = 0.031, *n* = 5). In the awake rat, all the evoked responses displayed features typical of climbing fiber field potentials. These included the following: (1) their pattern of response to a paired pulse test, when two supramaximal stimuli were delivered at interstimulus intervals ranging from 30 to 120 ms, the second response always exhibited a reduction in size, and there was a progressive increase in recovery with increased interstimulus intervals (*n* = 10 rats; Fig. 2; Armstrong, 1968; Armstrong and Harvey, 1968); and (2) trial-by-trial fluctuations in response size and a progressive increase in amplitude with increasing stimulus intensity, reaching a plateau at ∼2.5 × T (Fig. 2*B*).

Additional evidence that the responses were climbing fiber in origin was obtained in three animals (GRB, GRC, GRF) in which single-unit and multiunit Purkinje cell activity was recorded at the same recording sites where the largest CFPs were evoked. Similar to the evoked field potentials, complex spikes were evoked at a latency of 12.07 ± 1.17 (*n* = 5; Fig. 2*C*). Also, under terminal anesthesia, the neurotoxin 3-AP, which is known to preferentially lesion the inferior olive (Seoane et al., 2005), was used in three animals to test whether this abolished the peripherally evoked CFPs. After ∼3 h (the time point when 3-AP selectively disrupts olive activity), the amplitude of the evoked response was significantly reduced, shown by a decrease to ∼40% of preinjection response levels (Fig. 2*D*, dashed blue line). Baseline levels were never reached because of a concomitant increase in oscillatory activity in the LFP power in the 200–300 Hz frequency range from 0.025 ± 0.001 mV^2^/Hz (−45.9 dB/Hz) at 0 min to 0.142 ± 0.023 mV^2^/Hz (−38.5 dB/Hz) at 195 min (*n* = 3, representing an approximately fivefold increase in power over the 3 h period after 3-AP treatment; Fig. 2*E* and example case in Fig. 2*F*, bottom). The increase in the power spectral density at 200–300 Hz presumably reflects increased pathophysiological activity of Purkinje cells caused by loss of their climbing fiber input (Cerminara and Rawson, 2004; Janahmadi et al., 2009). In two control experiments in which a saline injection was made, the evoked CFPs remained similar in size over the same time period (Fig. 2*D*, solid black line), and there was no significant change in power of the LFP signal (Fig. 2*E*,*F*, top). Together, these data provide evidence that the evoked responses were likely to be climbing fiber in origin and generated as a result of transmission in ascending SOCPs arising from the ipsilateral hindlimb. However, a mossy fiber-related component cannot be entirely excluded.

### Changes in size of evoked response during rearing behavior

**Figure 3.**
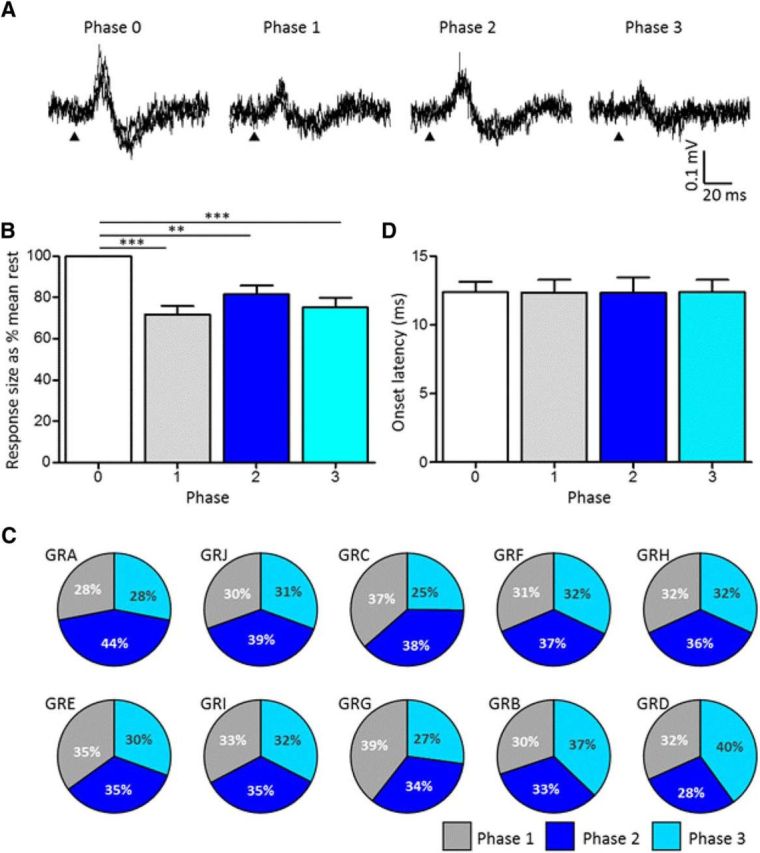
Changes in transmission in climbing fiber paths during rearing behavior. ***A***, Example case (animal GRA) showing evoked CFPs during rest and three different phases of rearing (3 sweeps superimposed in each panel). Phase 0, Rest; phase 1, rearing up; phase 2, upright position; and phase 3, rearing down. Arrowhead indicates time of ipsilateral hindlimb stimulation. ***B***, Bar graphs showing average size of evoked responses normalized to rest for the three phases of rearing (mean ± SEM; one-way ANOVA, *F*_(3,39)_ = 11.14, *p* < 0.0001; Tukey's multiple comparison test, **p < 0.01, ***p < 0.001, *n* = 10 rats). ***C***, Pie charts showing for each animal response size evoked during the three different phases of rearing expressed as a proportion of the total percentage change in response size relative to rest. Cases are shown in order of the largest to the smallest proportion in phase 2. ***D***, Same as ***B*** but onset latency (mean ± SEM).

As a first step toward testing our hypothesis that the gating serves to regulate the times when unpredictable signals are relayed to the cerebellum, we developed a method to probe pathway excitability during rats' natural exploratory behavior of rearing. Rearing activity was divided into three phases: phase 1, rearing up; phase 2, an upright position when the rat is standing on its hind legs; and phase 3, rearing down, when the rat lowers its upper body back to floor level. In each recording session, the hindlimb stimulus was delivered pseudorandomly during these different phases of rearing. Responses evoked in each phase were averaged across a number of trials, and the results were compared with the mean size of response obtained in the same recording session when the animal rested quietly without overt movement (phase 0). Figure 3*A* shows data from one animal (rat GRA), typical of the results as a whole. Compared with rest, responses evoked during phases 1 and 3 were reduced in size and also during phase 2 but to a lesser extent. In this example, the reductions in response size relative to rest were statistically highly significant in all three phases of rearing (one-way ANOVA, *p* < 0.0001, *F*_(3,135)_ = 21.77; Dunnett's multiple comparison test comparing each phase; *p* < 0.01 for all comparisons).

Data for all 10 cases are summarized in Figure 3*B*, which shows there was a statistically significant reduction in response size relative to rest during all three phases of rearing (one-way ANOVA, *F*_(3,39)_ = 11.14, *p* < 0.0001; Tukey's multiple comparison test, **p < 0.01, ***p < 0.001, *n* = 10). Overall, the evoked responses in phase 1 were similar in size to those evoked in phase 3, which were smaller than those evoked in phase 2 (on average, 71, 73, and 87%, respectively, of mean response size evoked at rest). Statistical analysis of the three different phases of rearing supports this general pattern (one-way ANOVA; *p* = 0.0001, *F*_(2,27)_ = 12.74; Tukey's multiple comparison test; phase 1 vs phase 2, *p* < 0.001; phase 1 vs phase 3, *p* > 0.05; phase 2 vs phase 3, *p* < 0.01). The general pattern of modulation during rearing was also evident when the analysis was restricted to the smallest number of available trials for a given phase, indicating that the rearing-related modulation could not be explained by variations in sample size between different phases of the movement.

To investigate whether there was any heterogeneity in pattern of gating between individual animals, the results for each animal are shown in Figure 3*C*. Each pie chart shows response size per phase of rearing expressed as a proportion of the total sum difference in response size relative to rest for all three phases (see Materials and Methods). For the majority of animals (7 of 10), the largest responses during rearing occurred when the animal was in the upright position (phase 2, dark blue). The exceptions were one animal (GRG) in which the largest responses occurred during rearing up (phase 1, gray) and two animals (GRB and GRD) in which the largest responses occurred during rearing down (phase 3, light blue). We were unable to identify any factors, such as differences in mediolateral location of recording site for animals GRB and GRD, to suggest why they might differ from the typical pattern, although one possibility that would require additional study to test is that there may have been differences in position of the implanted stimulus electrodes. This could have resulted in activation of different parts of the hindlimb. The gating associated with rearing may operate only on a selected set of afferent inputs related to that movement and its sensory consequences. Therefore, differences in location of the hindlimb stimulation may account for the heterogenous patterns of gating in these two cases.

With regard to animal GRG, this was one of two cases in which the highest stimulus intensity was used (3.1 × *T*; see Table 1). The other case in which the maximum stimulus intensity was used (rat GRC) had the next largest mean size of response in phase 1, suggesting that higher strengths of stimulus can to some extent override the modulation during this phase of rearing. This implies that different mechanisms may be responsible for the gating of responses in specific phases of rearing. This was supported by the additional finding that, for phases 2 and 3, a positive correlation was evident between mean size of response and stimulus intensity (phase 2, Pearson's *r* = 0.46, *p* = 0.183; phase 3, Pearson's *r* = 0.77, *p* = 0.009), but no such relationship was observed for phase 1 (Pearson's *r* = −0.003, *p* = 0.994). In contrast to the systematic differences in size of evoked CFP, the onset latency of the responses remained similar across rest and the three different phases of rearing activity (Fig. 3*D*).

### Central gating of SOCPs

**Figure 4.**
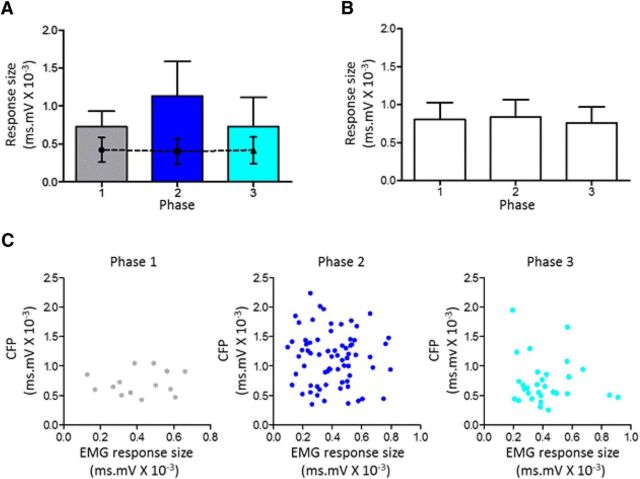
Changes in evoked CFPs are not related to simultaneously evoked muscle reflex. ***A***, Representative case (animal GRA) plotting for the three phases of rearing average size of CFP (bars) and EMG (dashed line) evoked by the same ipsilateral hindlimb stimulation (mean ± SEM in both plots). The phase-related variations in mean size of evoked CFPs were statistically significant (one-way ANOVA, *p* < 0.0001, *F*_(2,112)_ = 13.41; Tukey's multiple comparisons test: phase 1 vs phase 2, *p* < 0.01 and phase 2 vs phase 3, *p* < 0.001). In contrast, the mean size of evoked EMG responses were not significantly different (one-way ANOVA, *p* = 0.877, *F*_(2,112)_ = 13.19). ***B***, Pooled data from six animals showing mean size of evoked EMG response relative to the three phases of rearing (mean ± SEM, one-way ANOVA, *p* = 0.971, *F*_(2,15)_ = 0.0298). ***C***, Scatter plots for one example case (animal GRA), showing trial-by-trial fluctuations in evoked CFP and EMG for the three phases of rearing (Pearson's correlation; phase 1, *r* = 0.156, *p* = 0.58; phase 2, *r* = −0.065, *p* = 0.59; phase 3, *r* = −0.113, *p* = 0.55).

One possible explanation for the phase-related changes in response size is that the efficacy of the peripheral stimulus varied systematically as a result of movement-related changes in the position of the hindlimb stimulating leads during rearing. If this was the case, it might be expected that EMG reflex responses evoked by the same peripheral stimulus would display a similar pattern of phase-related variation in amplitude. In six animals, we monitored EMG responses evoked in hindlimb muscles (onset latency, 7.3 ± 0.9 ms, *n* = 6). The latency of these responses were in good agreement with activation of hindlimb muscle reflexes evoked by low threshold (Aβ) caliber afferent stimulation (Woolf and Swett, 1984). A representative example is shown in Figure 4*A*, which shows that, on average, there was no statistically significant difference in size of evoked EMG response during the three different phases of rearing (Fig. 4*A*, dashed line). In contrast, the CFPs evoked by the same peripheral stimulus displayed a phase-related modulation in response size consistent with the results as a whole (Fig. 4*A*, bars). The relative constancy of the evoked EMG responses during rearing is also evident when the data are pooled for all six animals (Fig. 4*B*). There was also no statistically significant correlation between trial-by-trial variations in EMG response size and simultaneously recorded CFPs for any of the phases of rearing in all six animals (Fig. 4*C*, phases 1–3, *p* > 0.05 in all 18 comparisons). It therefore seems reasonable to assume that the phase-related variations in size of the evoked CFP during rearing were mainly attributable to central modulation of SOCP transmission rather than any movement-related variations in the effectiveness of the stimulus.

### Changes in response size over time

**Figure 5.**
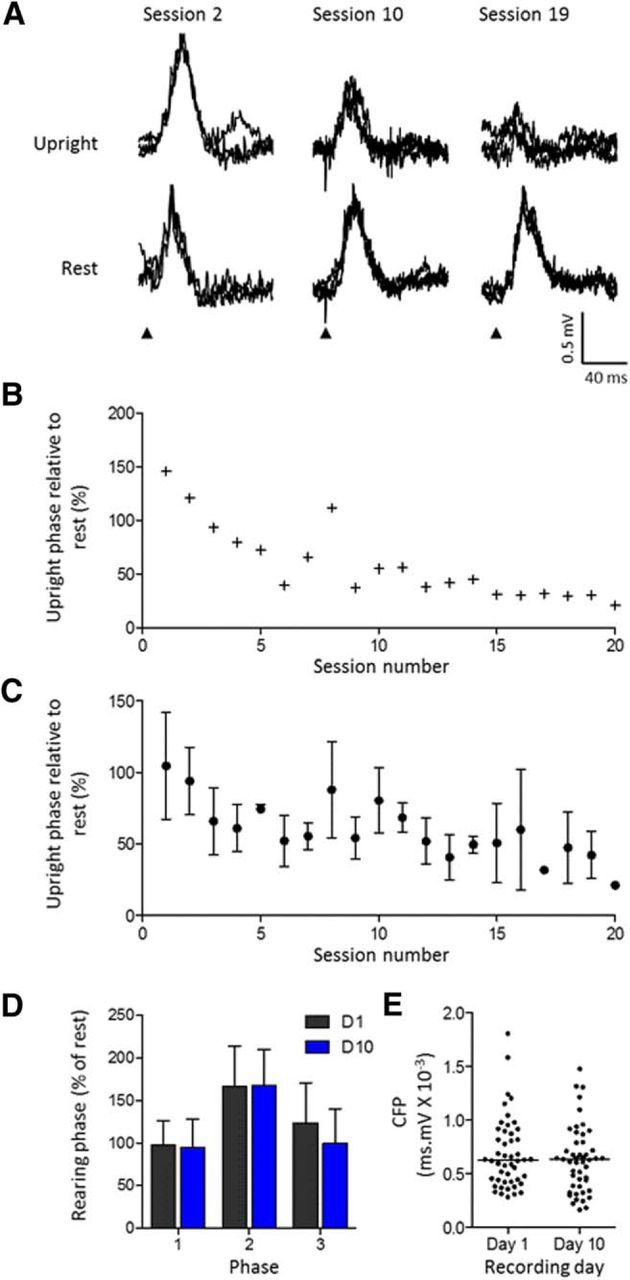
Changes in transmission as a stimulus becomes predictable. ***A***, Example traces (3 sweeps superimposed) obtained from one case (animal GRJ) showing CFPs evoked during the upright phase of rearing (top row) and CFPs evoked in the same recording session but during rest (bottom row). Examples are taken from three different time points during training (from recording sessions 2, 10, and 19, on days 1, 5, and 10, respectively). ***B***, For the same animal, mean size of evoked response relative to rest plotted as function of recording session number (Pearson's correlation, *r* = −0.828, *p* < 0.0001). ***C***, Same as ***B*** but pooled data from three animals showing mean response size ± SD (Pearson's correlation, *r* = −0.770, *p* < 0.0001). ***D***, Individual case showing mean size of evoked response expressed as a percentage of rest for the three phases of rearing for days 1 and 10 (D1 and D10). Same recording site on the 2 different days and responses evoked by pseudorandom presentation of the ipsilateral hindlimb stimulus (mean ± SD, two-way ANOVA interaction, *F*_(2,192)_ = 0.86, *p* = 0.424; day, *F*_(1,192)_ = 1.13, *p* = 0.289; phase, *F*_(2,192)_ = 27.31, *p* < 0.0001). ***E***, Distribution of size of individual responses evoked at the same recording site during rest on days 1 and 10 (unpaired *t* test, *p* = 0.286).

To investigate the possibility that the gating of transmission during behavior is adaptable, in three animals (GRH, GRI, GRJ), we delivered the peripheral stimulus in each recording session during the same phase of rearing (upright position, phase 2). Figure 5, *A* and *B*, displays the results for an individual animal and plots for each recording session the mean size of the response evoked in phase 2 expressed as a percentage of the mean size of response evoked at rest during the same session. On average, CFPs evoked during phase 2 of rearing showed a progressive reduction in size over the course of the whole recording period (10 d), reaching a minimum in recording session 20 (Fig. 5*B*, Pearson's *r* = −0.82, *p* < 0.0001). The negative correlation was also apparent when the data from all three animals were pooled (Fig. 5*C*, Pearson's *r* = −0.77, *p* < 0.0001). To control for the possibility that the progressive reduction in response size may be attributable to other factors, such as progressive changes in the characteristics or position of the recording electrode over time, in one animal, responses were evoked pseudorandomly during rearing over a similar time period as the test animals. In this case, there was no statistically significant change in size of evoked CFP relative to rest for any of the different phases of rearing (Fig. 5*D*, for phases 1–3, *p* > 0.05, two-way ANOVA), nor was there any significant change in size of evoked CFP during rest (Fig. 5*E*, unpaired *t* test, *p* = 0.286). Thus, the progressive reduction in size of the evoked CFP when the peripheral stimulus was delivered repeatedly during the same phase of rearing is unlikely to be attributable to systematic changes in recording conditions over time. Therefore, we conclude that the changes over time were attributable to modification of the central mechanism that regulates pathway transmission.

## Discussion

Key findings from the present study include the following: (1) CFPs evoked by transmission in hindlimb SOCPs can be recorded in the cerebellar cortex of rats during their natural exploratory behavior of rearing; (2) when the responses were evoked pseudorandomly during different phases of rearing, they were always smaller in size than those evoked at rest, and there were also phase-related differences: responses evoked in phase 1 (rearing up) and phase 3 (rearing down) were usually smaller than those evoked in phase 2 (standing upright on both hindlimbs); and (3) when the stimulus was repeatedly delivered only during phase 2, over a period of 1–2 weeks, the responses became progressively smaller. Together, these results indicate that transmission of input to climbing fibers terminating in the hindlimb C1 zone are significantly modulated during rearing behavior in rats and that the pattern of modulation can be modified by experience.

### Characterization of evoked field potentials in the awake rat

The evoked cerebellar fields and their response characteristics were similar to climbing fiber responses previously reported in the rat and cat (Armstrong and Harvey, 1968; Apps et al., 1995, 1997; Koutsikou et al., 2015). Additional evidence that the responses were attributable to activation of climbing fibers was demonstrated by the effect of 3-AP and also the finding that complex spikes could be evoked at a similar latency. It therefore seems reasonable to conclude that the fields were mainly, if not exclusively, the result of transmission in SOCPs.

The stimulus intensities used primarily activate Aβ caliber peripheral afferents (Armstrong and Harvey, 1968; Leicht et al., 1973; Ekerot et al., 1987, 1991; Apps et al., 1997). As a result, the present responses are most likely the result of transmission in afferents that relay innocuous sensory signals from cutaneous, muscle and joint receptors from the hindlimb. Consistent with previous studies (Apps et al., 1995, 1997), some evidence was also obtained to suggest that stimulus strength can influence the temporal pattern of modulation. The highest stimulus intensity used (3.1 × *T*) resulted in larger evoked responses in phase 1 but not other phases of rearing, suggesting a degree of functional heterogeneity in the SOCPs under study. Rather than the rearing-related modulation arising from the operation of a single modulatory influence, this suggests that two (perhaps more) mechanisms are in action: (1) one sensitive to stimulus strength and (2) one that is not. The hindlimb C1 zone receives sensory information via two classes of SOCP: the dorsal funiculus and ventral funiculus SOCPs. Both differ in their number of spinal and brainstem relays and are compound systems containing multiple subpaths (Ekerot et al., 1979; Ito, 1984). There is therefore ample scope within the pathways under study for differential modulation. For example, various inhibitory mechanisms are known to reduce transmission in climbing fiber pathways, including ascending mechanisms at the level of the dorsal column nuclei (Lidierth, 1991) and descending mechanisms from the red nucleus and cerebellar nuclei inhibiting the olive (Hesslow and Ivarsson, 1996; Horn et al., 1996; Kim et al., 1998). In particular, classical conditioning experiments indicate that the inhibitory nucleo-olivary pathway may be important in regulating transmission of the unconditioned stimulus to the cerebellum relayed via the climbing fiber system (Andersson and Hesslow, 1987; Hesslow and Ivarsson, 1996; Bengtsson et al., 2004).

One difference from previous reports of evoked climbing fiber fields was the bimodal distribution of response durations with corresponding differences in latency to peak. However, no relationship between short- or long-duration responses and any of the parameters studied in the present experiments could be found. Longer-duration fields imply less synchronous recruitment of Purkinje cells (cf. Apps and Lee, 2002). One possibility is that the longer-duration responses with a later latency to peak may involve short and long loop circuits, e.g., convergence of a direct spinal pathway and an indirect loop via the hindlimb region of the somatosensory cortex (cf. Morissette and Bower, 1996; Proville et al., 2014). However, given that the temporal pattern of modulation during rearing for longer-duration responses was indistinguishable from the pattern obtained for short-duration responses, this would suggest that indirect pathways targeting the hindlimb C1 zone in the cerebellar cortex are subject to a similar (or common) pattern of gating as more direct hindlimb SOCPs.

An additional highly consistent finding was that the onset latency of the responses evoked by hindlimb stimulation were significantly shorter in the awake versus anesthetized rat. A similar phenomenon has not been reported for forelimb SOCPs. However, barbiturate (sodium pentobarbital) anesthesia has been shown to increase onset latencies compared with ketamine in the rat (Morissette and Bower, 1996). Also, barbiturates can severely depress transmission of mossy fiber pathways (Eccles et al., 1968), while enhancing the amplitude of climbing fiber responses (Gordon et al., 1972). Because barbiturate is an allosteric modulator and agonist for GABA receptors, presumably the longer latency of climbing fiber fields evoked by hindlimb stimulation in the anesthetized preparation is attributable to greater susceptibility at one or more relay sites along the ascending pathway to barbiturate inhibition, slowing transmission.

### Transmission is modulated during rearing activity

The finding that there was an overall reduction in evoked response size during rearing compared with rest indicates a behavior-dependent decrease in transmission in SOCPs. This is in good agreement with previous studies, mainly in cats, that have shown movement-related reductions in transmission of sensory signals relayed via SOCPs (Gellman et al., 1985; Lidierth and Apps, 1990; Apps et al., 1995; Horn et al., 1996; Apps and Lee, 1999; Pardoe et al., 2004; Ozden et al., 2012) but extends this phenomenon to include natural exploratory activity in rats and transmission in hindlimb pathways. Previously, little information was available on hindlimb SOCPs during behavior. Short-latency hindlimb and forelimb climbing fiber pathways involve different SOCPs and target different somatotopically organized regions of the cerebellar cortex (hindlimb and forelimb receiving areas). The finding that gating of transmission also occurs in hindlimb pathways in the rat adds weight to the notion that this is a general phenomenon of SOCPs that is conserved across species.

We found that the largest decreases in hindlimb SOCP transmission usually occurred during rearing up (phase 1) and rearing down (phase 3), which coincide with active movement. These are times when phasic EMG activity occurs in hindlimb muscles. In contrast, during the upright phase (phase 2), little or no active limb movement occurs, but rather tonic hindlimb muscle activity is required for maintaining posture and balance, often combined with head movements associated with scanning the environment. However, it cannot be excluded that the modulation is instead, or at least in part, dependent on limb position (cf. Horn et al. 1996). Although there were exceptions, the size of the evoked CFPs during phase 2 were generally similar in size to those evoked at rest, suggesting the associated SOCPs were open for transmission during upright posture when the hindlimbs are in a fully extended position.

### Longer-term modification of cerebellar input and possible functional significance

The present results indicate that pathway transmission can be modified after stimulation delivered during the same phase of rearing behavior over the course of many days. Such findings are consistent with previous studies that used classical conditioning of eye or forelimb reflexes to show transmission in olivo-cerebellar paths can be altered by associative learning (Hesslow and Ivarsson, 1996; Apps and Lee, 2002; Rasmussen et al., 2008). The present results extend these observations by demonstrating that the pattern of modulation can be dynamically altered during natural exploratory behavior.

The prevailing view is that climbing fibers relay error signals to the cerebellar cortex whenever there is a mismatch between intended and achieved movement to drive long-term changes in Purkinje cell synaptic efficacy underlying motor learning (Yeo and Hesslow, 1998). The notion of error signaling is also incorporated into forward models of motor control (Wolpert et al., 1995). An internal representation of a movement (located within the cerebellum) integrates sensory and motor signals to generate predictions about the outcome of a movement, and sensory error-related climbing fiber signals are thought to be used to update the model (Wolpert and Miall, 1996; Kitazawa et al., 1998).

The pattern of gating during rearing might then reflect the changing usefulness of climbing fiber signals to update the internal model (cf. Pardoe et al., 2004). Pathway transmission is normally reduced during phases 1 and 3 of rearing when self-generated afferent signals occur, which are predictable (non-error signals) and therefore presumably of limited value for updating the model. In contrast, the same pathways are more open for transmission during the upright phase (phase 2) when the rat needs to be vigilant to predatory attack. However, when the stimulus is delivered repeatedly during phase 2, over many trials, the modulation adapts to gate out a signal that has become expected. The results are therefore consistent with the hypothesis that the gating of transmission in SOCPs serves to prevent predictable sensory signals being forwarded to the cerebellum via the climbing fiber system.
